# The conserved Fanconi anemia nuclease Fan1 and the SUMO E3 ligase Pli1 act in two novel Pso2-independent pathways of DNA interstrand crosslink repair in yeast

**DOI:** 10.1016/j.dnarep.2013.10.003

**Published:** 2013-12

**Authors:** Y. Fontebasso, T.J. Etheridge, A.W. Oliver, J.M. Murray, A.M. Carr

**Affiliations:** a Genome Damage and Stability Centre, University of Sussex, Brighton, East Sussex BN1 9RQ, UK; b Breakthrough Breast Cancer Research Centre, The Institute of Cancer Research, 237 Fulham Road, London SW3 6JB, UK

**Keywords:** ICL, Genetic screen, Synthetic array, Epistasis, *Schizosaccharomyces pombe*, Cisplatin

## Abstract

DNA interstrand cross-links (ICLs) represent a physical barrier to the progression of cellular machinery involved in DNA metabolism. Thus, this type of adduct represents a serious threat to genomic stability and as such, several DNA repair pathways have evolved in both higher and lower eukaryotes to identify this type of damage and restore the integrity of the genetic material. Human cells possess a specialized ICL-repair system, the Fanconi anemia (FA) pathway. Conversely yeasts rely on the concerted action of several DNA repair systems. Recent work in higher eukaryotes identified and characterized a novel conserved FA component, FAN1 (Fanconi anemia-associated nuclease 1, or FANCD2/FANCI-associated nuclease 1). In this study, we characterize Fan1 in the yeast *Schizosaccharomyces pombe*. Using standard genetics, we demonstrate that Fan1 is a key component of a previously unidentified ICL-resolution pathway. Using high-throughput synthetic genetic arrays, we also demonstrate the existence of a third pathway of ICL repair, dependent on the SUMO E3 ligase Pli1. Finally, using sequence-threaded homology models, we predict and validate key residues essential for Fan1 activity in ICL repair.

## Highlights

• Characterization of FANCD2/FANCI-associated nuclease 1 (Fan1) in the model organism *Schizosaccharomyces pombe*.
• Fan1 is a key component of a previously unidentified ICL resolution pathway in *Schizosaccharomyces pombe* acting in parallel to Pso2
• Demonstrate the existence of a third sub-pathway of ICL repair dependent on the SUMO E3 ligase Pli1.
• The nuclease and SAP DNA binding domains are essential for Fan1 activity in ICL repair.

## 1 Introduction

Interstrand cross-links (ICLs) represent a particularly insidious threat to genomic stability. These adducts create covalent bonds linking the two DNA strands in a duplex, generating an abnormal structure that poses a physical obstacle to the progression of cellular machinery like DNA replisomes [1,2]. The mechanisms underlying the response to ICLs in unicellular organisms depend on components involved in many of the major DNA repair pathways: nucleotide excision repair (NER), base excision repair (BER), mismatch repair (MMR), post-replication repair (PRR, comprising translesion synthesis, TLS) and homologous recombination (HR) [2]. Conversely, only a few proteins have been identified as specific to the response to ICLs. In *Saccharomyces cerevisiae*, Pso2/Snm1 has been identified as a key player in the response to interstrand cross-linking agents [3–5]. A role for Snm1/Pso2 has been postulated where its exonuclease activity resects the DNA flanking the ICL to facilitate TLS or homologous recombination [6,7]. Although little is known about the resolution of DNA ICLs in the fission yeast *Schizosaccharomyces pombe*, the corresponding Pso2 nuclease has been similarly shown to be required for normal resistance to ICL-inducing agents [8].

In higher eukaryotes, multiple DNA repair pathways are also involved in the resolution of ICLs, albeit the existence of the specialized Fanconi anemia (FA) pathway [9] marks a significant difference compared to the yeasts. The current model for the involvement of the FA pathway in ICL repair is as follows: the ICL is recognized by FANCM-FAAP24 bound to the recently discovered MHF complex [9–11]. FANCM-FAAP24-MHF recruits a downstream E3 ubiquitin ligase complex known as the “FA core complex”, which in turn monoubiquitinates FANCD2 and FANCI on chromatin [9,12]. FANCD2-FANCI then recruits further downstream factors and interacts with HR and TLS proteins, finally facilitating HR-dependent ICL repair [9]. It is also proposed that a parallel crosstalk with S-phase checkpoint proteins mediates and coordinates ICL repair with other DNA damage response mechanisms [9].

Recent work in higher eukaryotes identified and characterized FAN1 (Fanconi anemia-associated nuclease 1, or FANCD2/FANCI-associated nuclease 1) [13–18]. Human FAN1 colocalizes to ICL-induced foci with and dependently on monoubiquitinated FANCD2, suggesting a role with the FA pathway. Defects in homologous recombination in FAN1-depleted cells suggest that this protein is involved in the HR processes linked to ICL repair. As DSB resection is not impaired in the absence of FAN1 and RAD51 foci persist in FAN1-depleted cells, it has been proposed that FAN1 may be required for late stages of HR-dependent repair [14,15]. A homolog of FAN1 is present in the fission yeast *S. pombe*, but not in the budding yeast *S. cerevisiae*. Thus, the appearance of FAN1 earlier than the FA core complex-dependent pathway on the evolutionary scale suggests that the role of this component is either functionally distinct from the canonical FA pathway of higher eukaryotes or is regulated by this pathway. For this reason, the study of Fan1 in *S. pombe* has the potential for revealing mechanisms of ICL repair in higher eukaryotes which act in parallel with, or are controlled by, the FA pathway.

In the present study, we investigate the function of *S. pombe* Fan1 (the gene is named *fan1* following the work discussed above) using standard and high-throughput genetics. We demonstrate that Fan1 is a novel component of a Pso2-independent ICL resolution pathway and genetically dissect these two pathways to assign epistatic relationships with known components of DNA damage repair pathways involved in ICL repair. Using high-throughput synthetic genetic arrays to explore genetic relationships in the response to ICL-inducing agents, we identify the existence of an additional ICL resolution pathway dependent on the SUMO E3 ligase Pli1. Finally, we identify key Fan1 residues necessary for its activity.

## 2 Material and methods

### 2.1 DNA damaging agents

UV irradiation was performed with a Stratagene^®^ Stratalinker^®^ using the settings (J/m^2^) indicated. Other drugs used, all from SIGMA^®^: methyl methanesulfonate (MMS), cat. no. 129925; cisplatin (*cis*-platinum(II)diammine dichloride, product no. P4394; mitomycin C (MMC), cat. no. M0503; HN1 (2-chloro-*N*,*N*-dimethylethylamine hydrochloride), product no. 24362; HN2 (mechlorethamine hydrochloride), product no. 122564; cycloheximide, product no. C7698 (100 mg/l from a 100 mg/ml stock in DMSO).

### 2.2 Strains

A list of all the strains used in this study is provided in supplementary Table 4. Details of the strain construction for specific mutants are given below.

### 2.3 *fan1*-d strain construction

Two independently-derived *fan1*-d mutants (both *fan1::kanMX*; kanMX confers resistance to the drug geneticin, or G418) have been analysed in parallel in this study. The first mutant (3909) was kindly donated by Professor Paul Nurse; the second mutant (14152) is derived from the Bioneer^®^ *S. pombe* deletion mutant library (http://pombe.bioneer.co.kr/). The two strains were verified by Southern blot. To allow a flexible and rapid series of genetic crosses between different deletion mutants, the original kanMX deletion cassettes in the 3909 and 14152 strains were replaced with a natMX6 deletion cassette, which confers resistance to nourseothricin [19]. The natMX6 null mutants derived from 3909 and 14152 were named 3909N and 14152N, respectively. These new mutants showed the same sensitivity to the drugs used in this study as the original 3909 and 14152 strains (data not shown). The two independently derived mutants always showed consistent sensitivity to the drugs tested. The 3909/3909N and 14152/14152N strains were used both in parallel for nearly all the experiments conducted, although in the interest of space only one of the two mutant is usually presented in the figures of this study. *fan1* mutants were created employing site-directed mutagenesis using a Stratagene QuikChange^®^ kit as described in [20] and Recombinase-Mediated Cassette Exchange (RMCE) as described in [21].

### 2.4 Spontaneous mutation rate assays

Single colonies were isolated on YEA from individual streaks. Eleven colonies from each strain were grown in 5 ml YE in individual tubes. Samples were incubated at 30 °C for 48 h to stationary phase. Cultures were serially diluted as follows: 10 μl saturated culture in 1 ml H2O; 10 μl of this dilution into 1 ml H_2_O. A 50 μl of this dilution were plated on YE-Agar (YEA) plates. A 50 μl of saturated culture were plated on YE-5-FOA (5-Fluoroorotic acid; Melford^®^ F5001) plates (0.1% final concentration). Plates were incubated for 3–4 days at 30 °C. Spontaneous mutation rates were calculated by the Lea-Coulson method of the median (Rosche and Foster, 2000; Foster, 2006).

### 2.5 *In vivo* survival assays: Spot tests

Strains were inoculated in 5 ml YE and grown at 30 °C o/n. 10^7^ cells from each logarithmically growing culture were harvested and resuspended in 1 ml water. Four serial 1/10 dilutions were prepared from each culture. A 10 ul were spotted onto YEA plates added with increasing doses of DNA damaging agents. All the spots were deposited in duplicates on the same plate to guarantee an internal control. Plates were incubated at 30 °C for 3 days. Images were acquired with a Syngene^®^ Ingenius^®^ apparatus.

### 2.6 *In vivo* survival assays: Survival curves

A 2 × 10^8^ cells grown to exponential phase were centrifuged and washed with PBS. Pellets were resuspended in 10 ml and split into five 2 ml aliquots in 15 ml tubes. Each drug dilution was inoculated into the 2 ml aliquoted cultures and tubes incubated at 30 °C with shaking for 1 h. Approximately 200 cells were plated onto YEA and grown at 30 °C for 3–4 days.

### 2.7 Automated Screening of the Bioneer deletion library

*Note*: all the parameters of the programs indicated below are detailed in the supplementary section. A loopful of query mutant (Q) was inoculated from a fresh patch into 15 ml YE + NAT and grown for at least 6 h. The above culture was poured into an empty PlusPlate^®^ (“Q bath”). Once thawed, library plates were replicated onto YEA PlusPlates^®^: four liquid 96-well plates combined onto one YEA PlusPlate^®^ (384 spots) [PROGRAM 1, TWICE PER ARRAY]. A 384 agar plate was build using the Q bath as a source (“Q YEA PlusPlates^®^”) [PROGRAM 2, TWICE PER ARRAY]. Cells were grown for 2–3 days at 30 °C (or until colonies are grown to satisfactory size). Each library was replicated to fresh YEA PlusPlates^®^ (“L YEA PlusPlates^®^”) [PROGRAM 3]. Mating: colonies were combined from the L and Q YEA PlusPlates^®^ onto ELN PlusPlates^®^ [PROGRAM 4, RUN TWICE PER ARRAY]. ELN PlusPlates^®^ were incubated at 25 °C for 4 days. YEA PlusPlates^®^ were incubated at 30 °C for 3 days: pictures were taken approximately every 12 h to monitor the fitness of the single mutants. Spore germination: colonies were replicated from ELN PlusPlates^®^ to YEA PlusPlates^®^ [PROGRAM 5] and incubated at 30 °C for 3 days. Selection 1: colonies were replicated from YEA PlusPlates^®^ to YE + GC PlusPlates^®^ [PROGRAM 5] and incubated at 30 °C for 2–3 days (or until colonies have grown to satisfactory size). Selection 2: cells were replicated from YE + GC PlusPlates^®^ to YE + GNC PlusPlates^®^ [PROGRAM 3] and incubate at 30 °C for 1–3 days. Pictures to assess the fitness of double mutants were taken at this stage every approximately 12 h.

For the assessment of resistance to DNA damaging agents, cells were replicated from YE + GNC PlusPlates^®^ to YE PlusPlates^®^ added with different concentrations of chosen DNA damaging agents [PROGRAM 3]. Plates were incubated at 30 °C for 2-4 days and pictures were taken approximately every 12 h. Pictures were taken using a Syngene^®^ Ingenius^®^ apparatus. Software used for colony size analysis: HT Colony Grid Analyser 1.1.0/1.1.7, Adobe^®^ Photoshop^®^ CS5 Extended, Microsoft^®^ Excel^®^ 2007/2010.

## 3 Results

### 3.1 The Fanconi anemia—Associated nuclease Fan1 is not involved in the suppression of DNA spontaneous mutation rate

Human FAN1 (also known as KIAA1018) has been shown to interact with MMR components such as MLH1, PMS1 and PMS2 [13,14,22]. Thus, we set out to test whether a similar scenario holds true for SpFan1, and whether this protein could be involved in the mismatch repair pathway. As we were unable to detect direct physical interactions of SpFan1 with other MMR components (data not shown), we performed a forward mutation assay in order to determine the rate of spontaneous mutation in *fan1*-deleted cells. In this system, the readout is the switch from uracil autotrophy to uracil heterotrophy. The estimated mutation rate during DNA replication in eukaryotic cells is lower than 1 mutation every 10^9^ bases [23], which would be undetectable by our current mutation assays. In *S. cerevisiae*, a mutation in the catalytic subunit of polymerase delta (Pol3-L615M) leads to a 7-fold increased spontaneous mutation rate with no measurable changes in other phenotypes monitored [24]. In our study, the background spontaneous mutation rate was therefore increased to detectable levels by using a strain harbouring the corresponding mutation in polymerase delta, Cdc6 (*cdc6-L591M*) [25].

In a *cdc6-L591M* background, the mutation rate is increased to approximately 1 in 10^6^ (Table 1; consistent with [25]). However, no significant increase in this spontaneous mutation rate was observed following concomitant deletion of *fan1*. These data argued against a direct involvement of SpFan1 in the MMR pathway.

### 3.2 Fan1 is a component of a Pso2-independent interstrand crosslink repair pathway

In order to determine whether SpFan1 could be involved in other pathways of DNA repair, we performed *in vivo* survival assays to assign genetic interactions between SpFan1 and known components of characterized repair pathways involved in the ICL response. To assess the response of *fan1*-d mutants to a variety of DNA lesions, the two SpFan1 deletion mutants 3909 and 14152 were initially back-crossed twice to a wild-type strain and five independent G418-resistant colonies were isolated and tested under increasing concentrations of various DNA damaging agents. All the *fan1* deletion isolates showed wild-type sensitivity to UV, camptothecin (CPT), methyl methanesulfonate (MMS) and hydroxyurea (HU) (data not shown). However, a subtle but reproducible sensitivity was observed when *fan1-d* cells were exposed to *cis*-platinum diammine-dichloride (cisplatin, CDDP) and mitomycin C (MMC). These drugs belong to a family of DNA damaging agents that induce covalent DNA interstrand cross-links [2]. The mild sensitivity towards ICL-inducing agents suggested that SpFan1 is implicated in ICL repair, but that its role overlaps with the function of other components of the DNA repair machinery.

**Fig. 1:**
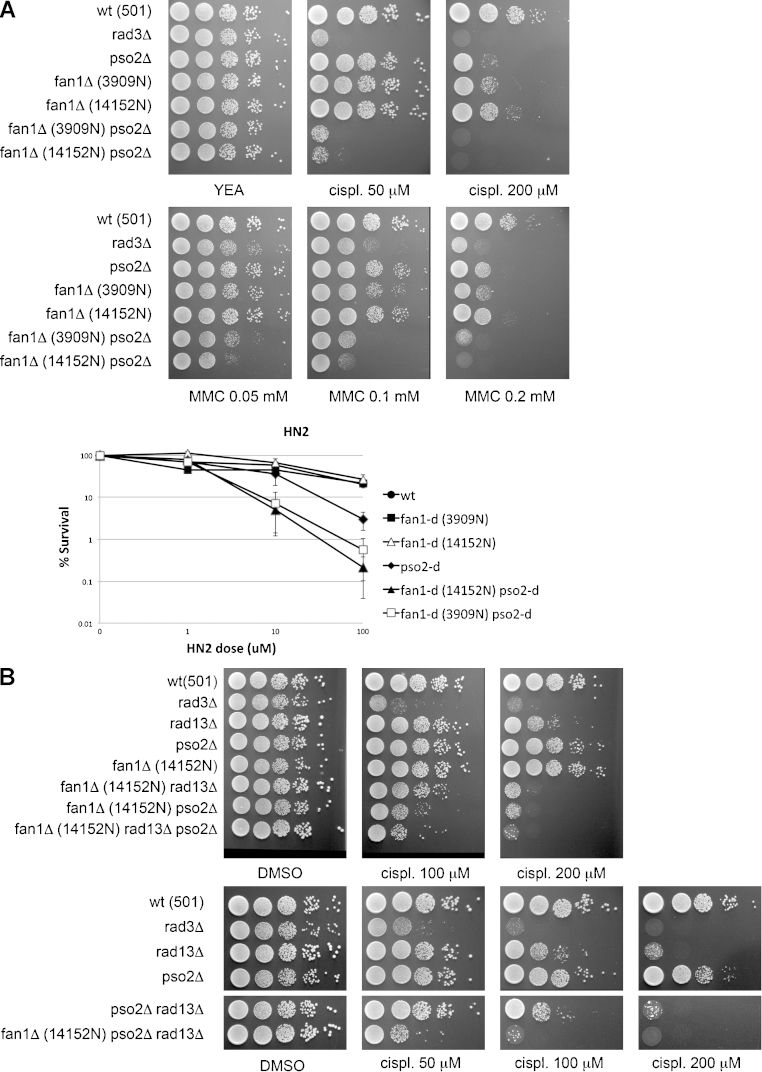

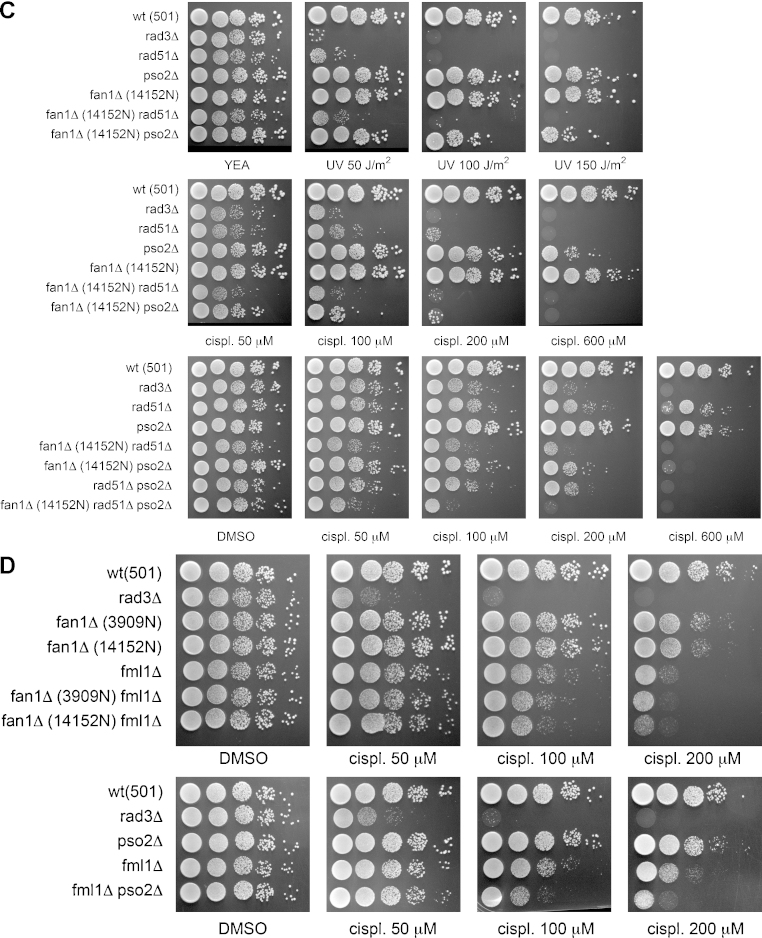
*Fan1 is a novel component of a pso2-independent interstrand crosslink repair pathway in S. pombe*. (A) Sensitivity of combinations of fan1 and pso2 deletion mutants to cisplatin and MMC. Top panel: logarithmically grown cultures were spotted in four 1:10 serial dilutions starting from 10^7^ cells (first spot on the left) on YEA plates containing the agents in the amount indicated. Bottom panel: due to the short half-life of HN2, sensitivity to this drug was assessed by exposing 4 × 10^7^ cells from logarithmically growing cultures to the indicated dose. Error bars represent the standard error of the mean of three independent experiments. (B) Sensitivity of combinations of *fan1*, *pso2* and *rad13* deletion mutants to cisplatin (*fan1*-d: 14152N background). (C) Sensitivity of combinations of *fan1*, *pso2* and *rad51* deletion mutants. (D) Sensitivity of combinations of *fml1* deletion mutants. *rad3*-d is used as a standard hypersensitive control for the efficacy of the agents used. UV treatment was included as a control for rad51 sensitivity. cispl, cisplatin; MMC, mitomycin C; HN2, bis(2-chloroethyl) methylamine; UV, ultra-violet irradiation.

To test this, the original 3909 and 14152 *fan1* null mutants were crossed with a series of deletion mutants of genes reported to be involved in the ICL resolution pathway, either in *S. pombe* or in the budding yeast *S. cerevisiae.* In *S. pombe*, the nuclease Pso2 (also known as Snm1 in *cerevisiae*) has been shown to be specifically required for normal resistance to ICL-inducing agents [8]. When exposed to increasing doses of cisplatin and MMC, the *fan1-d pso2-d* double mutant showed a dramatic reduction in viability compared to the corresponding single mutants or the wild-type (wt) control strain (Fig. 1A, left panel). In order to confirm that SpFan1 is specifically involved in ICL repair, cell survival assays were repeated for *fan1-d* and *pso2-d* using bis(2-chloroethyl)methylamine (HN2, mechloretamine), an agent shown to generate a higher proportion of DNA interstrand cross-links compared to cisplatin [2]. When exposed to increasing concentrations of HN2, *fan1*-deleted cells showed a marked decrease in viability only when combined with *pso2* deletion (Fig. 1A, right panel). As a further control, the same experiment was conducted in the presence of HN1 (2-dimethylaminoethylchloride hydrochloride), a mono-functional nitrogen mustard which does not form ICLs [26]. None of the strains treated with this agent, including the double mutant *pso2-d fan1-d*, showed any sensitivity to this agent (data not shown).

Taken together, these data confirm that SpFan1 is a novel component of the DNA repair pathway that specifically acts to repair cross-links linking covalently the two strands of a DNA molecule, and that SpFan1 and SpPso2 act in parallel pathways or subpathways.

### 3.3 The NER nuclease Rad13 is involved only in the pso2-dependent ICL repair pathway

ICL repair mechanisms in lower and higher eukaryotes have proven to be elusive due to the intersection of different DNA repair pathways. In *S. cerevisiae*, components of the nucleotide excision repair (NER), post-replication repair (PRR) and homologous recombination (HR) pathways have all been implicated in the resolution of interstrand cross-links [2]. To test whether Fan1-dependent ICL repair intersects with these pathways, a series of double and triple mutants were created and tested for sensitivity to cisplatin.

*rhp18*, the fission yeast gene encoding the homolog of *S. cerevisiae* Rad18 involved in post-replication repair[2], displayed hypersensitivity to cisplatin to concentrations as low as 50 μM (supplementary Fig. 1). The combination of the *fan1* and *rhp18* mutations did not display increased sensitivity to cisplatin, whereas a mild but reproducible increase in sensitivity was observed when *pso2*-*d* was combined with *rhp18*-*d* (supplementary Fig. 1). This indicates that Rhp18 is involved in the resolution of ICL adducts in a step that is common to the Fan1 pathway. The deletion of the nuclease Exo1 displayed no significant sensitivity to ICL-inducing agents, and only a marginal increased sensitivity in combination with pso2-d, fan1-d (double mutants) or with pso2-d fan1-d (triple mutant) (data not shown).

Of all the mutants tested (including *msh2*-d, *chk1*-d and *cds1*-d), *rad13* deletion showed the most dramatic reduction in viability, compared to the wt strain, when exposed to cisplatin (1b, top panel). SpRad13 (HsXPG, ScRad2) is a nuclease centrally involved in the NER pathway that is required for the initial incision at early steps of ICL repair in *S. cerevisiae* [2]. Interestingly, *rad13*^*XPG*^ null mutant sensitivity was significantly further increased when combined with a deletion of the gene coding for Fan1, but not when combined with deletion of the gene coding for Pso2 (Fig. 1B, compare top and bottom panel). Interestingly, the triple mutant *fan1*-d *pso2*-d *rad13*-d (14152N background) phenocopied the *fan1*-d *pso2*-d strain (Fig. 1B, top and bottom panels). The same pattern of sensitivity was observed for the 3909N background (data not shown). These data suggest that, in *S. pombe*, Rad13^XPG^ is involved in the resolution of DNA interstrand cross-links in a Pso2- but not in the Fan1-dependent pathway. We decided to test whether a similar differential involvement is true also for SpRad16, the homolog of the DNA repair endonuclease XPF in human. The deletion of *rad16* caused a dramatic sensitivity to cisplatin (supplementary Fig. 2). However, no further increased sensitivity was noticed in the double mutants *pso2*-d *rad16*-d or *fan1*-d *rad16*-d, nor in the triple mutant *pso2*-d *fan1*-d *rad16*-d (supplementary Fig. 2). This result suggests that rad16 is epistatic to both the Fan1- and the Pso2-dependent pathways.

### 3.4 Homologous recombination is required for ICL resolution downstream the Fan1- and Pso2-dependent pathways

Homologous recombination has been shown to be involved in the repair of ICLs in the budding and the fission yeast [2,8]. Rad51 protein is required for most recombination events in yeast [27]. The deletion of both *fan1* and *rad51* led to a marked drop in viability compared to wild-type and single mutants when cells were exposed to cisplatin, but not when they were exposed to UV (Fig. 1C), which consistent with a predominant involvement for Rad51 but not Fan1 in the response to UV-induced damage. In contrast, the deletion of both *pso2* and *rad51* did not increase the sensitivity to cisplatin compared to *rad51* single mutant cells (Fig. 1C, bottom panel). Interestingly, the triple deletion of the genes coding for Fan1, Rad51 and Pso2 resulted in the most dramatic decrease in viability compared to all the combinations of mutants tested (Fig. 1C, bottom panel). These data suggest a crucial role for Rad51 in the resolution of ICLs outside the Pso2 and Fan1 pathways. The notable difference in sensitivity between the combinations of *fan1*-*d rad51-d* and *pso2-d rad51-d* double mutants further suggests differential extents for the involvement of Rad51-dependent processes in the Pso2 and Fan1 pathways of ICL resolution.

### 3.5 The conserved Fanconi anemia component Fml1 acts in a Pso2-independent ICL resolution pathway

Fml1 is the *S. pombe* homolog of the human FANCM helicase/translocase, a component of the Fanconi anemia pathway [28,29]. Fml1 has been previously shown to be required for wild-type resistance to interstrand cross-linking agents such as cisplatin [30]. Recent work on the homolog Mph1 in *S. cerevisiae* indicates that Mph1 and Pso2 act in independent pathways of ICL resolution upon exposure to HN2 [31]. To test whether the same scenario holds true in *S. pombe*, we created combined double mutants of *fml1*-d and *pso2*-d or *fan1*-d and assessed the sensitivity of these mutants to cisplatin. Whereas the combination of *fml1*-d and *fan1*-d did not increase the sensitivity to the drug compared to the single mutants, the concomitant deletion of *fml1* and *pso2* showed a more accentuated sensitivity (Fig. 3D). This data suggests that, in parallel with the situation in the budding yeast, the conserved Fanconi anemia component Fml1 and the nuclease Pso2 act on independent pathways in response to resolution of DNA interstrand adducts.

### 3.6 The nuclease and the SAP DNA binding domain are required for Fan1 activity

**Fig. 2:**
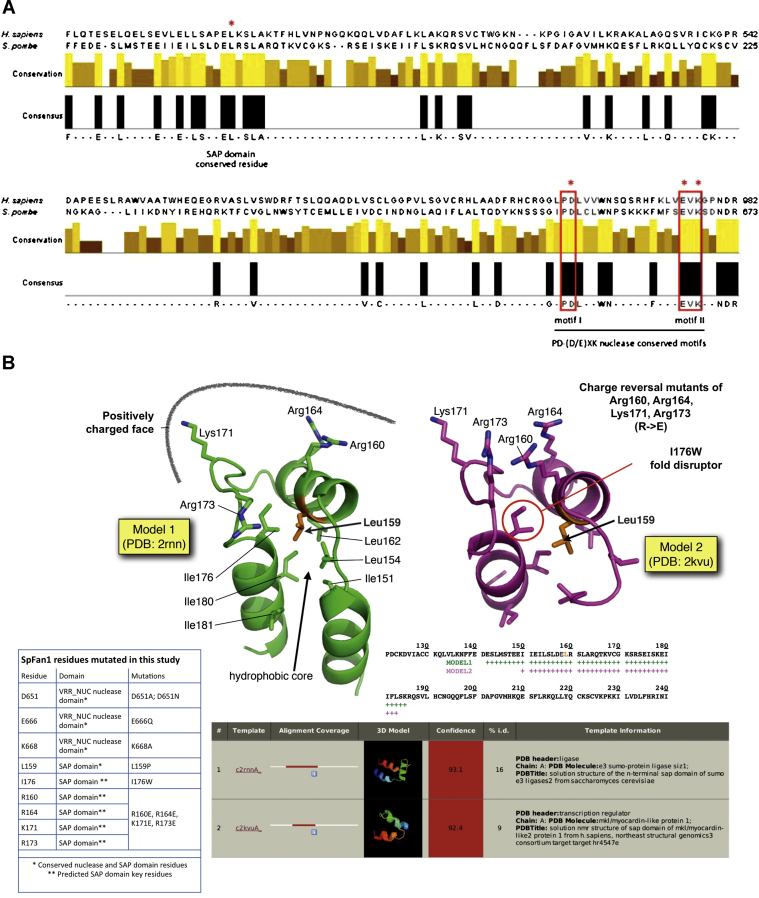

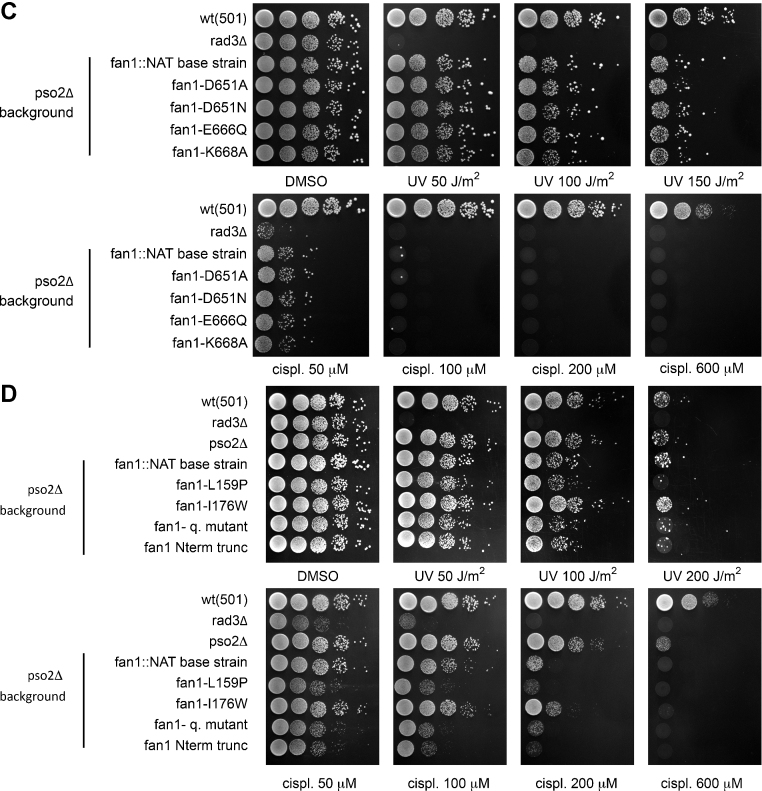
*The nuclease and the SAP domains of Fan1 are required for wild-type resistance to cisplatin*. (A) Amino acid sequence alignment between HsFAN1 and SpFan1. Manually annotated ClustalW2 alignment (http://www.ebi.ac.uk/Tools/clustalw2/index.html). The boxed regions indicate the conserved PD-(D/E)-XK nuclease motif [33]. Asterisks indicate the residues mutated in our study (Leu159, Asp651, Glu666, Lys668 in the *S. pombe* homolog). These, plus additional mutants are listed in Fig. 3B (inset table). (B) Phyre2 sequence-threaded models of the spFAN1 SAP domain. Molecular ‘cartoon’ representations of the structural models based on PDB templates 2rnn and 2kvu. Key amino acids are additionally show in stick representation. The extent, quality and detail of each model is indicated by the inset amino acid sequence alignment and associated Phyre2 summary table. (C) Sensitivity of point mutations in the conserved residues of the nuclease domain to cisplatin and UV. A *pso2*-d background was used in order to compare the effect of the mutations to the hypersensitive double mutant *fan1*-d *pso2*-d. Logarithmically grown cultures were spotted in four 1:10 serial dilutions starting from 10^7^ cells (first spot on the left) on YEA plates containing the agents in the amount indicated. *rad3*-d is used as a standard hypersensitive control for the efficacy of the agents used. UV, ultra-violet irradiation; cispl, cisplatin; q. mutant, *fan1*-R160E R164E K171E R173E; N-term trunc, N-terminal truncation mutant d. Sensitivity of point and truncation mutants in the SAP domain to cisplatin. As described under (C).

Previous work has indicated the presence of two conserved domains, shared between human and *S. pombe* Fan1 (Fig. 2A): a SAP-type DNA binding motif (SAF-A/B, Acinus and PIAS) thought to be involved in chromosomal reorganization [32] and a VRR_nuc (Virus-type Replication-Repair Nuclease) domain [13], which is associated with DNAses involved in DNA repair and is characterized by a relatively conserved PD-(D/E)XK motif [33,34]. We decided to test whether these domains were required for normal functionality of SpFan1. From amino-acid sequence alignments [13] three residues within the *S. pomb*e VRR_nuc catalytic motif – Asp651, Glu666, Lys668 – were selected for mutagenesis (Fig. 2A); D651A, D651N, E686Q, K668A. For the SAP domain, as only a single residue was strongly conserved between the human and the *S. pombe* homologs (Leu159) [13], we decided to generate sequence-threaded homology models, using the Phyre2 webserver (http://www.sbg.bio.ic.ac.uk/phyre2) to identify alternative amino acids to target for mutagenesis. Using two models, based on separate templates (PDB: 2rnn; 2kvu), we were able to identify a positively-charged face, comprised of amino acids Arg160, Arg164, Lys171, and Arg173 (Fig. 2B). We therefore designed speculative charge-reversal mutants in this region to disrupt any potential protein-DNA interactions; R160E, R164E, K171E, R173E. Using site-directed mutagenesis we generated single and multi-point mutants in both the VRR_nuc and SAP domain of spFAN1, then used recombinase-mediated cassette exchange [20] to introduce them into a *pso2*-deleted base strain, and tested them for sensitivity to UV and cisplatin. In addition, L159P and I176W mutants were designed to specifically perturb/disrupt the overall fold of the SAP domain, as well as a deletion mutant removing the entire SAP domain (N-term trunc; removing the first 193 amino acids of spFAN1).

As expected, the *pso2-d fan1::NAT (null)* double mutant base strain displayed a marked hypersensitivity when exposed to doses of cisplatin as low as 50 μM (Fig. 2C). All three nuclease domain mutants, Fan1-D651A, Fan1-E666Q and Fan1-K668A, phenocopied the *pso2 fan1* double null mutant when exposed to either UV or cisplatin (Fig. 2C). The substitution of D651 with the structurally similar, but formally non-charged, residue asparagine (Fan1-D651N) showed the same hypersensitivity as *fan1-D651A* (Fig. 2C). Similarly, the SAP domain mutants L159P, the quadruple charge-reversal mutant *fan1-R160E/R164E/K171E/R173E* and the N-terminal truncation mutant all phenocopied the *pso2 fan1* double null mutant (Fig. 2D). Taken together, these data suggest that both the VRR_nuc nuclease domain and the SAP DNA binding domain are essential for SpFan1 activity *in vivo*. Interestingly, the potential fold-disrupted mutant (*fan1-I176W*) appeared to be less sensitive than the other SAP domain mutants (Fig. 2D), suggesting that disrupting the corresponding alpha-helix affects Fan1 protein activity to a lesser extent.

### 3.7 High-throughput screens of synthetic genetic arrays identify a role for Pli1 in ICL resolution

**Fig. 3:**
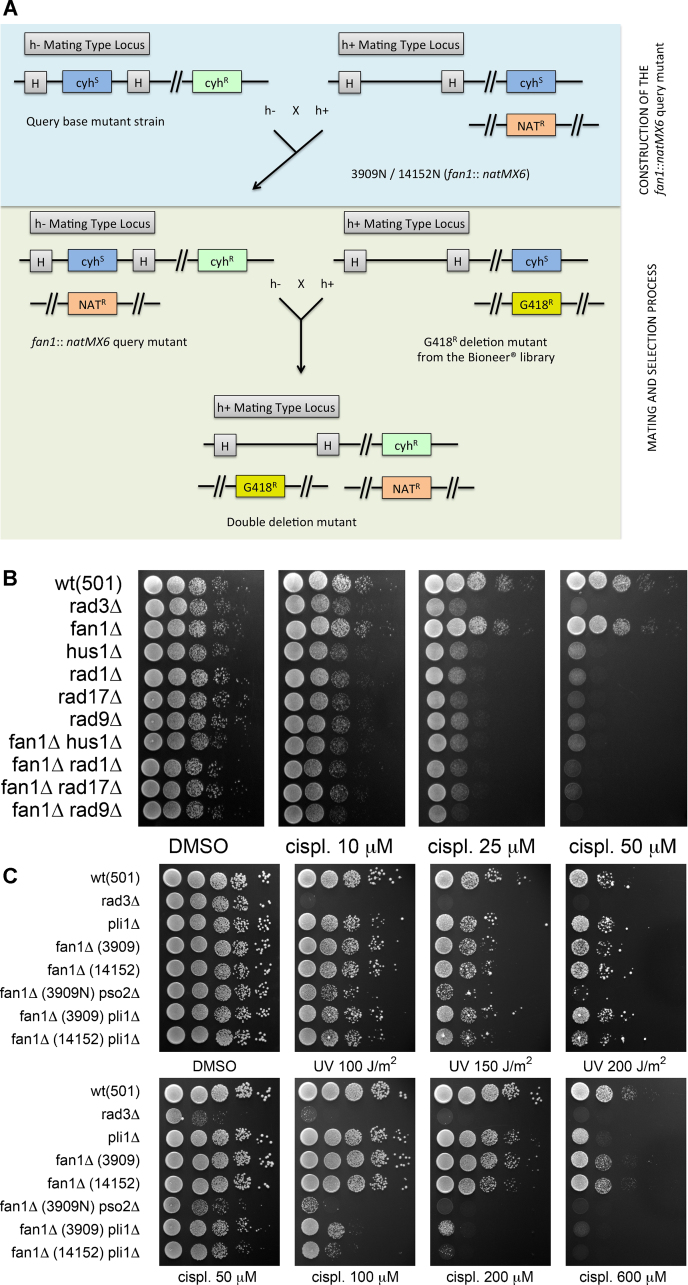
*The PEM-2 screen identifies a novel Pli1-dependent pathway of ICL repair*. (A). Schematic representing the marker selection process throughout the PEM-2 high-throughput screen. The PEM-2 (*Pombe* Epistatic Mapper—2) approach is based on recessive resistance to the drug cycloheximide. Step1 (blue panel): construction of the *fan1*::*natMX6* query mutant. Step 2 (green panel): screen of the Bioneer^®^ deletion mutant library. Mating and selection procedures ensure the maintenance of the three markers *NAT*^*R*^, *G418*^*R*^ and *cyh*^*R*^ (at the native locus), conferring to the final double deletion mutant resistance to nourseothricin, geneticin and cycloheximide, respectively. See supplementary section and [35] for further details. (B). Sensitivity to cisplatin of the combination of mutants *hus1*, *rad1* and *rad17* with *fan1*. Logarithmically grown cultures were spotted in four 1:10 serial dilutions starting from 10^7^ cells (first spot on the left) on YEA plates containing the agents in the amount indicated. *rad3*-d is used as a standard hypersensitive control for the efficacy of the agents used. The double mutants tested in this and the above experiments were derived from independently constructed single deletion mutants. *fan1*-d: 3909 and 14152 backgrounds. UV, ultra-violet irradiation; cispl, cisplatin. (C) Sensitivity to UV and cisplatin of the combination of *pli1* and *fan1* null mutants. As described under (B). (For interpretation of the references to color in this figure legend, the reader is referred to the web version of this article.)

In order to detect genetic interactions between *fan1* and other components of DNA metabolism, we set up a high-throughput genetic screen by constructing synthetic genetic arrays (SGAs) using the PEM-2 (*pombe* epistatic mapper 2) as described in [35]. Firstly, the screen involved the generation of arrays of haploid double mutants created by crossing a *fan1*-deleted query strain with 2034 individual null mutants from the Bioneer^®^ V2 deletion library (Fig. 3A and supplementary Fig. 3). The resulting double mutants were initially grown on standard media and the fitness of each double mutant colony assessed by computational analysis of colony size and classified according color-coded categories of deviation from the median colony size following the criteria (see supplementary Fig. 5). The full list of disruptants showing synthetic lethal/sickness and alleviating interactions with the *fan1*-d background is provided in supplementary Tables 1–3.

The double mutants were subsequently transferred to plates supplemented with increasing concentrations of cisplatin. The hypersensitivity to cisplatin exposure was again assessed by computational analysis of colony size. Briefly, synthetic genetic arrays of double null mutants were assessed for consistent, significant and progressive reduction of colony size upon increasing concentrations of cisplatin when compared to the median colony size of the population of double mutants growing on the same plate. A full description of the method applied is detailed in the supplementary section.

By intersecting the data from three independent screens, six candidates showed a progressive and consistent reduction in colony size upon increasing concentrations of cisplatin (Table 2). The presence of the DNA repair nuclease Rad13 in the list validated of the methodology, as the deletion of this gene already showed synergistic hypersensitivity to cisplatin when combined with *fan1* deletion (Fig. 1B).

Because this initial analysis was performed on the double mutants, it does not preclude that the hypersensitivity to cisplatin is due solely to the single mutation from the deletion library, and not to its combination with *fan1*-d. Thus, to assess whether the hypersensitivity shown in the screen represented synergistic hypersensitivity, and to provide a further validation of the methodology, double disruptants were recreated from independently derived mutants and tested by employing standard *in vivo* survival assays.

SpRad1 and SpHus1 are part of the 9-1-1 clamp complex, which play crucial roles in checkpoint activation following DNA damage [36,37]. SpRad17 acts as a clamp loader for the trimeric complex [37]. Consistently, all these three highly correlated factors were pulled out in the screen. SpRad9, third component of the 9-1-1 complex, was absent in the library of deletion mutants tested. When tested for sensitivity to cisplatin, both the *fan1*-d mutants 3909N and 14152N showed a strong hypersensitivity when combined with *rad1-d*, *hus1-d* or *rad17-d* (Fig. 3B). However, the single mutants were similarly highly sensitive, indicating an epistatic interaction between these checkpoint components and *fan1*. To determine whether the same occurs for the third component of the 9-1-1 complex SpRad9, independently derived double mutants *fan1*-d *rad9*-d were constructed and tested by *in vivo* survival assays. Consistently with a common role as part of the 9-1-1 heterotrimer, *rad9*-d phenocopied *hus1*-d and *rad1*-d, either as a single mutant or in combination with *fan1*-d (Fig. 3B).

Intriguingly, *fan1*-d *pli1*-d was also pulled out as a hypersensitive double deletion mutant. Pli1 is a SUMO (small ubiquitin-related modifier) E3 ligase that has been associated with DNA repair, although its roles have not yet been fully elucidated [38]. When tested using *in vivo* survival assays, independently constructed *fan1*-d *pli1*-d mutants (3909 or 14152 background) showed hypersensitivity to cisplatin compared to the wild-type, *fan1*-d and *pli1*-d strains (Fig. 3C). This increased sensitivity is dramatic following exposure to cisplatin and absent upon UV irradiation, indicating that the two proteins are required in response to the formation of a significant amount of DNA interstrand cross-links.

Taken together, these findings confirm that the application of the computational analysis of colony size to the high-throughput screen for sensitivity to cisplatin is an effective methodology, as it facilitated the identification of the involvement of the SUMO E3 ligase Pli1 in the resolution of DNA interstrand cross-links.

### 3.8 Further exploration of genetic relationships in the Pso2-independent ICL repair pathway

As our high-throughput computational approach proved to be effective in identifying new factors acting in a parallel pathway with Fan1 in response to ICL exposure, we adopted the same approach to identify potential genetic interactions triggered by cisplatin exposure in the absence of the *pso2*-dependent ICL responses. Similarly to the methodology described above, we assessed the reduction of colony size in haploid double mutants generated by crossing a *pso2*-deleted query mutant with a series of deletion mutants included in the Bioneer^®^ V2 deletion library. A selection of candidates, presented in Table 3, showed progressive dramatic sensitivity in two independent screens as a consequence of exposure to increasing concentrations of cisplatin.

Interestingly, the 9-1-1 protein Hus1 and the clamp loader Rad17 were again identified as hypersensitive mutants, confirming the importance of these components in response to ICL. Similarly to the screen with the *fan1*-deleted query mutant, we also pulled out Rad1 in one replica of the screen, but as the sensitivity was not evident in the second replica, this candidate was not included in the final list. The reason for the lack of significant sensitivity to cisplatin in the second replica is unknown. However, as our previous experiments showed clearly that *rad1* null mutant is hypersensitive to cisplatin, we classify this as experimental noise, likely due to cross-contamination with other strains, or due to the rise and over-growth of a cisplatin-resistant strain within the colony. All the mutants pulled out as hits in the *pso2*-deleted cisplatin screen were subsequently re-made using the single mutants present in the deletion library. A further independent test for cisplatin sensitivity validated the results obtained from the screen (supplementary Fig. 4). Interestingly, all the null mutants identified in this branch of the screen were not epistatic with Pso2 (supplementary Fig. 4).

## 4 Discussion

The data presented in this study substantiate a conserved role for FAN1 in the resolution of interstrand cross-links across eukaryotes. The prospective role for SpFan1 in the resolution of this type of adducts was confirmed not only by the sensitivity of the null mutant to a series of ICL-inducing agents, but also by the dramatic increase in sensitivity to the same agents when the deletion of *fan1* and *pso2* were combined (Fig. 1A).

### 4.1 Genetic dependencies in the novel SpFan1-dependent pathway of ICL resolution

As the nuclease SpPso2 was previously identified as a key component of the ICL response in *S. pombe* [8], our results suggested that SpFan1 is a key component of a novel pathway or sub-pathway of ICL repair, acting in parallel with the one dependent on SpPso2. The initial systematic genetic analysis with other double and triple deletion mutants of candidate genes identified only one other dramatic increased combined sensitivity to interstrand cross-linkers: the *fan1*-d *rad13*-d double mutant (Fig. 1B). SpRad13 (homolog of Rad2Sc and XPGHs) is a core nuclease involved in the double incision step of the nucleotide excision repair pathway, 3′ to the lesion [39]. The finding that the combination of *pso2*-d and *rad13*-d did not lead to increased sensitivity to cross-linkers (Fig. 1B) places this nuclease uniquely in the Pso2-dependent pathway of ICL resolution.

Consistently with other studies in eukaryotes, the E3 ubiquitin ligase Rhp18 was found to be required for wild-type resistance to interstrand cross-links (supplementary Fig. 1 and [8,40–42]. However, only the combined deletion *rhp18*-d *pso2*-d showed increased sensitivity to cisplatin compared to the most sensitive single mutant (supplementary Fig. 1), suggesting that Rhp18 is required for the Fan1- and not for the Pso2-dependent pathway. In this context, the involvement of SpRhp18 in ICL repair might echo what has been proposed in *S. cerevisiae*, where Rad18 would be implicated in controlling DNA synthesis at late stages of ICL processing in conjunction with Rad6. However, further work is needed to support this hypothesis.

A fourth gene deletion found to confer sensitivity to cisplatin was *rad51*-d, coding for the homolog of the recombination protein Rad51 [43,44]. Interestingly, but not unexpectedly, the deletion of *rad51* showed increased sensitivity following exposure to cisplatin when combined with either *fan1* or *pso2* deletion, compared to the single mutants (Fig. 1C). Rad51 has been already implicated in ICL repair in the fission yeast [45]. The data presented in this study suggests that Rad51 would be involved in both the Fan1- and Pso2-dependent pathways (Fig. 1C). In particular, the hypersensitivity of *rad51*-d seems to be more dramatic in combination with *fan1*-d, suggesting that the Fan1 pathway would rely on Rad51-dependent homologous recombination to a lesser extent when compared to the Pso2 pathway. It is also interesting to note that the triple deletion strain *fan1*-d *pso2*-d *rad51*-d appears to be even more sensitive compared to any of the cognate strains (Fig. 1C). This observation might suggest that Rad51 has additional functions in ICL response that are independent of Fan1 and Pso2. Alternatively, it may reflect the fact that the agents used do not exclusively induce ICLs.

Finally, the systematic genetic analysis led to the discovery of the epistatic relationship between Fan1 and Fml1, the FANCM helicase/translocase homolog in *S. pombe*. To our knowledge, prior to this study Fml1 was the only conserved component of the FA pathway in the fission yeast. Thus, following the work presented here, the epistasis with Fan1 in ICL resolution suggests that these two enzymes may represent a prototypical FA pathway in *S. pombe*.

### 4.2 The molecular function of Fan1 in ICL resolution

Very limited assumptions can be made about the function of SpFan1 in this novel pathway of ICL repair. Data from the analysis of SpFan1 point mutants lead to the conclusion that at least three key residues in the VRR_nuc nuclease domain are required for the function of the protein in the ICL response: D651, E666 and K668 (Fig. 2C). In human cells, point mutations in the corresponding residues D960, E975, K977 compromise Fan1 exo- and endonucleolytic activities [13,15,17]. Although biochemical studies with *S. pombe* Fan1 have not been performed, it is reasonable to speculate that SpFan1 acts in the ICL resolution pathway as a nuclease. The depletion of the conserved SAP domain in Fan1 (L159P, quadruple mutant and N-term truncation mutant) significantly affects its function. This would be consistent with a role for the conserved SAP domain in mediating contact with the damaged substrate DNA, as has been proposed for other proteins possessing this domain [32].

From the limited data available thus far, it is not possible to assign a specific function to Fan1 in processing ICL lesions. However, it is interesting to note that the nuclease Rad13^XPG^ has been found to be non-epistatic with Fan1 and epistatic with Pso2 (Fig. 1B). It is tempting to speculate that another nuclease may be needed in the Fan1 pathway to cover the role exerted by Rad13^XPG^ in the Pso2 pathway. Rad13^XPG^ (homolog of ScRad2/HsXPG) is a crucial component of the nucleotide excision repair pathway, involved in the endonucleolytic incision 3′ to the adduct [39]. Consistently with its role in NER, it has been proposed that, in mammalian cells. XPG would be involved in the unhooking step of the ICL pathway (3′ of the lesion), although the finding that XPG-depleted cells are only mildly sensitive to ICL agents suggests that other nucleases, such as MUS81-EME1, may also play a prominent, potentially redundant, role [46,47]. It is possible that in *S. pombe*, as well as in higher eukaryotes, multiple nucleases are involved in the endonucleolytic unhooking step of ICL resolution. In the light of the biochemical studies with mammalian FAN1 [13–15], it can be suggested that SpFan1 may be implicated in this reaction, either 3′ or 5′ to the ICL. It would be interesting to test the *in vitro* and *in vivo* requirements for various nucleases that may be predicted to be involved at this stage in the fission yeast, including Mus81/Eme1, the Rad16^XPF^, Rad13^XPG^ and Fan1 itself.

The biochemical data for human FAN1 indicates that this enzyme may be additionally involved in other stages of ICL repair. Firstly, its exonuclease activity might be required in the trimming of the unhooked ICL. Secondly and more importantly, the significant defects shown for FAN1- depleted cells at late stages of homologous repair indicate that this nuclease might be predominantly involved in the processing of recombination intermediates generated by treatments with DNA cross-linkers [14,15]. The data presented in this study do not allow further conclusion on a similar role for SpFan1.

### 4.3 The role of SUMOylation in the DNA interstrand cross-link pathway

**Fig. 4:**
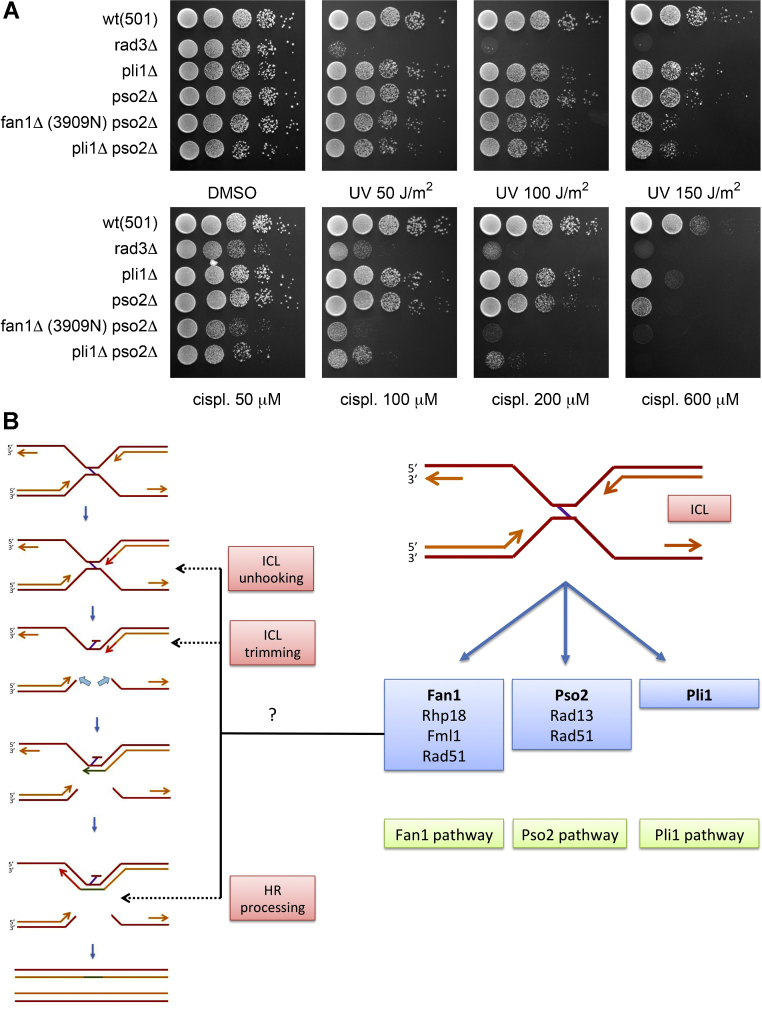
*Pli1 acts on a pathway of ICL repair distinct from the fan1- and the pso2-dependent systems*. (A) Sensitivity of *pli1*-deleted mutants combined with deletions of *fan1* and *pso2*. *fan1*-d: 3909 background. Logarithmically grown cultures were spotted in four 1:10 serial dilutions starting from 10^7^ cells (first spot on the left) on YEA plates containing the agents in the amount indicated. *rad3*-d is used as a standard hypersensitive control for the efficacy of the agents used. Abbreviations used: UV, ultra-violet irradiation; cispl, cisplatin. (B) Proposed schematic of ICL resolution in *S. pombe*. The components of the various DNA repair pathways are shown in the relevant boxes, as assigned from the genetic analysis presented in this study. Left panel: possible roles for Fan1 in the Fan1-dependent resolution pathway. For simplicity, only the double fork model of ICL resolution [48] is shown.

An interesting outcome of the cisplatin high-throughput screen was the identification of the increased sensitivity of the combined *fan1*-d *pli1*-d mutant compared to the parental single mutants (Table 2 and Fig. 3C). SpPli1 is a ligase involved in the post-translational conjugation of small proteins named SUMO (small ubiquitin-related modifiers). Although the exact significance of this conjugation (SUMOylation) is still debated, it is clear that this class of reversible modifications plays a widespread and important role in the regulation of eukaryotic biological processes including DNA repair (reviewed in [38]). In the context of this study, the hypersensitivity of the *fan1*-d *pli1*-d mutant to cisplatin highlights a crucial involvement of SUMOylation in an ICL resolution pathway distinct from the one in which SpFan1 is implicated. The additional epistasis analysis presented in Fig. 4A indicates that this Pli1-dependent ICL resolution pathway is likely defining an additional, third way of addressing this type of adduct in *S. pombe*. Our study thus demonstrates an unprecedented role for SUMOylation in the resolution of interstrand cross-links in *S. pombe* which might be conserved in higher eukaryotes.

### 4.4 Multiple pathways or sub-pathways of ICL resolution in *Schizosaccharomyces pombe*

Based on the data presented here, it is possible to delineate the participation of some of the components of the DNA repair machinery in the resolution of interstrand cross-links in the fission yeast *S. pombe*. A schematic is presented in Fig. 4B, where the known Pso2 pathway of ICL resolution is paralleled by the newly identified Fan1 and Pli1 pathways.

Fig. 4B (left panel) shows the possible molecular roles for Fan1 in the ICL resolution pathway, as discussed in the previous section.

## 5 Conclusions

This study profited from the use of the fission yeast *S. pombe* as a model organism to investigate the role of novel components acting in response to DNA interstrand cross-link formation, one of the most insidious threats posed to genomic stability. DNA interstrand cross-linking agents are amongst the most widely used treatments of a wide range of cancers. Studies in mammalian systems stemming from the outcome of the present work may thus ultimately translate to an increased efficacy of the current clinical options, for instance by targeting parallel ICL repair pathways in ICL repair-deficient tumours to selectively aggravate the cytotoxicity of the current oncological treatments.

## Work individual contributions

Conception and design: Carr AM, Murray JM, Fontebasso Y.

Experimental execution: Fontebasso Y (standard genetic assays and setup of high-throughput genetic screens), Etheridge TJ (site-directed mutagenesis and generation of mutant strains), Oliver AW (generation of sequence-threaded homology models and design of mutants).

Writing, review, and/or revision of the manuscript: Fontebasso Y (writing), Carr AM, Oliver AW (review/revision)

## Conflict of interest statement

None.

## Figures and Tables

**Table 1:** *Fan1 is not involved in the suppression of spontaneous mutation rate.* Spontaneous forward mutation rate of fan1-d mutants in cdc6+ and cdc6-L591M backgrounds. Data from three independent experiments. For each strain, 11 colonies were grown to saturation at 30 °C for 48 h. Fluctuation analysis was performed as described in Section 2.

	Mutation rate/cell division					
	Experiment 1	Experiment 2	Experiment 3	Average	Standard error	Fold elev.
*cdc6+*	8.09E − 10	5.27E − 10	5.65E − 10	6.33E − 10	8.85E − 11	1
*cdc6+ fan1*-d (3909)	9.98E − 10	8.45E − 10	9.17E − 10	9.20E − 10	4.42E − 11	1
*cdc6+ fan1*-d (14152)	6.49E − 10	5.74E − 10	5.02E − 10	5.75E − 10	4.24E − 11	1
*cdc6*-L591M	2.08E − 06	1.08E − 05	2.82E − 06	5.25E − 06	2.81E − 06	1
*cdc6*-L591M *fan1*-d (3909)	2.55E − 06	6.35E − 06	3.37E − 06	4.09E − 06	1.16E − 06	1
*cdc6*-L591M *fan1*-d (14152)	1.90E − 06	2.93E − 06	2.99E − 06	2.61E − 06	3.54E − 07	0

**Table 2:** Double mutants (background: fan1-d mutant) that showed progressive increased sensitivity to cisplatin in all the three independent screens. Gene IDs, Bioneer^®^ plate reference, gene names and descriptions are extracted from the strain list provided with the Bioneer^®^ deletion mutant haploid set.

Gene ID	Bioneer^®^ plate Ref.	Gene name	Description
SPAC1687.05	V2-05-A01	pli1	SUMO E3 ligase Pli1
SPAC1952.07	V2-05-B05	rad1	Checkpoint clamp complex protein Rad1
SPBC3E7.08c	V2-13-C04	rad13	DNA repair nuclease Rad13
SPAC20G4.04c	V2-19-C02	hus1	Checkpoint clamp complex protein Hus1
SPAC9E9.14	V2-28-D07	vps24	Vacuolar sorting protein Vps24
SPAC14C4.13	V2-30-G05	rad17	RFC related checkpoint protein Rad17

**Table 3:** Double mutants (background: pso2-d mutant) that showed progressive increased sensitivity to cisplatin in two independent screens. Gene IDs, Bioneer^®^ plate reference, gene names and descriptions are extracted from the strain list provided with the Bioneer^®^ deletion mutant haploid set.

Gene ID	Bioneer^®^ plate Ref.	Gene name	Description
SPAC24B11.12c	V2-05-D11		P-type ATPase
SPBC3E7.08c	V2-13-C04	rad13	DNA repair nuclease Rad13
SPAC11E3.04c	V2-16-E10	ubc13	Ubiquitin conjugating enzyme Ubc13
SPAC20G4.04c	V2-19-C02	hus1	Checkpoint clamp complex protein Hus1
SPCC23B6.05c	V2-27-B11	ssb3	DNA replication factor A subunit Ssb3
SPAC4D7.06c	V2-27-E12		Siroheme synthase
SPAC14C4.13	V2-30-G05	rad17	RFC related checkpoint protein Rad17
