# Corrigendum to “The conserved Fanconi anemia nuclease Fan1 and the SUMO E3 ligase Pli1 act in two novel Pso2-independent pathways of DNA interstrand crosslink repair in yeast” [DNA Repair 12 (December (12)) (2013) 1011–1023]

**DOI:** 10.1016/j.dnarep.2014.11.003

**Published:** 2014-12

**Authors:** Y. Fontebasso, T.J. Etheridge, A.W. Oliver, J.M. Murray, A.M. Carr

**Affiliations:** a Genome Damage and Stability Centre, University of Sussex, Brighton, East Sussex BN1 9RQ, UK; b Breakthrough Breast Cancer Research Centre, The Institute of Cancer Research, 237 Fulham Road, London SW3 6JB, UK

The authors have mentioned the following corrections:

Page 1013: “(Fig. 1A, left panel)” should read “(Fig. 1A, top panel)”
Page 1013: “(Fig. 1A, right panel)” should read “(Fig. 1A, bottom panel)”
Page 1016: “fml1 and pso2 showed a more accentuated sensitivity (Fig. 3D)” should read “fml1 and pso2 showed a more accentuated sensitivity (Fig. 1D)”

The authors would also like to acknowledge Dr John Rouse for his contribution at the initial stage of the project: “We thank John Rouse for helpful advice on the project and for sharing unpublished results”.

The authors would like to apologise for any inconvenience caused.

