# Supplementary material for: The conserved Fanconi anemia nuclease Fan1 and the SUMO E3 ligase Pli1 act in two novel Pso2-independent pathways of DNA interstrand crosslink repair in yeast
Source: DNA Repair (Amst). 2013 Dec;12(12):1011–23. doi: 10.1016/j.dnarep.2013.10.003 (PMC4045212; doi:10.1016/j.dnarep.2013.10.003)
Supplement: Supplementary file 1 [file mmc1.docx]

# Supplementary material

## Supplementary figures


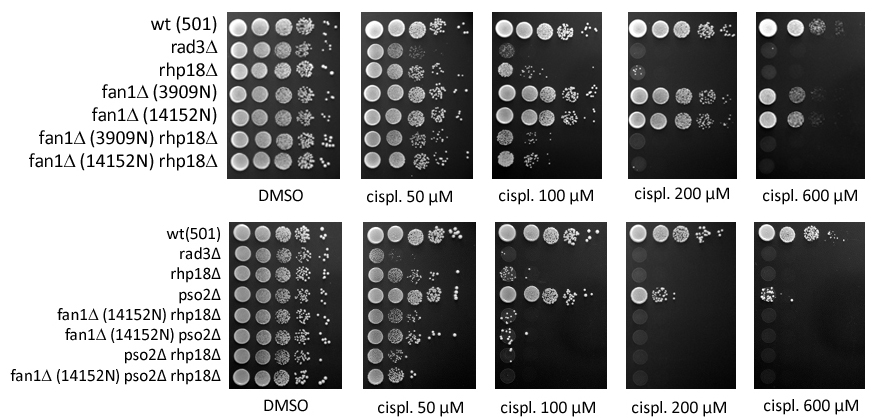


**Supplementary figure 1 | Sensitivity of *rhp18*-deleted mutants combined with deletions of *fan1* and *pso2*.** *fan1*-d: 3909N background. Logarithmically grown cultures were spotted in four 1:10 serial dilutions starting from 10^7^ cells (first spot on the left) on YEA plates containing the agents in the amount indicated. *rad3*-d is used as a standard hypersensitive control for the efficacy of the agents used. Abbreviations used: UV, Ultra-Violet irradiation; cispl, cisplatin.


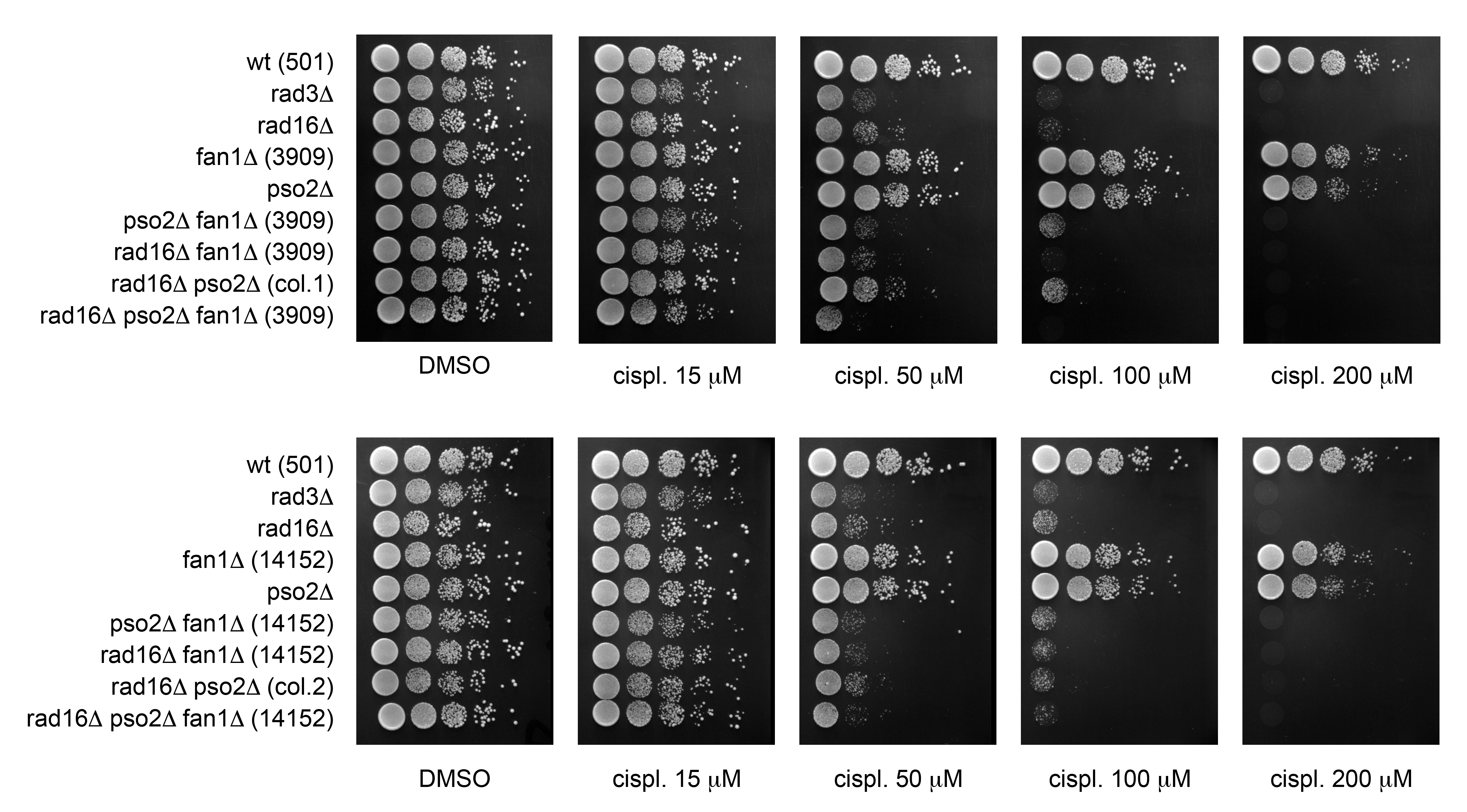


**Supplementary figure 2 |** **Sensitivity of *rad16*-deleted mutants combined with deletions of *fan1* and *pso2*.** Logarithmically grown cultures were spotted in four 1:10 serial dilutions starting from 10^7^ cells (first spot on the left) on YEA plates containing the agents in the amount indicated. *rad3*-d is used as a standard hypersensitive control for the efficacy of the agents used. Abbreviations used: cispl, cisplatin.


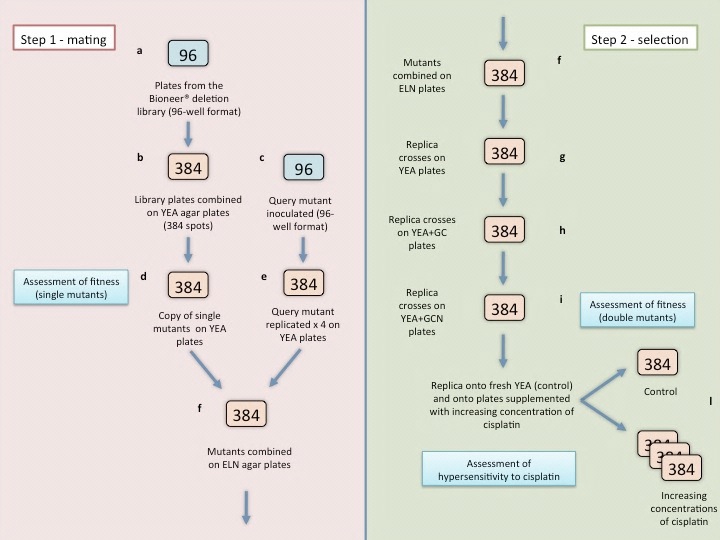


**Supplementary figure 3 | Schematic overview of the pinning procedures to construct the synthetic genetic arrays used in the present work.** The screen was performed in a 384-pin format. Four 96-well plates from the Bioneer® library were combined on one YEA 384-spot agar plate (a-b). The query mutant was inoculated in parallel from a 96-well to a 384-spot format (c-e). The screen was repeated for at least three times from at least two independent query mutant isolates. All the replicas in steps g-I were carried out with a Singer® RoToR© HDA station by use of short 384 pins.

*Intended for color reproduction on the Web only*


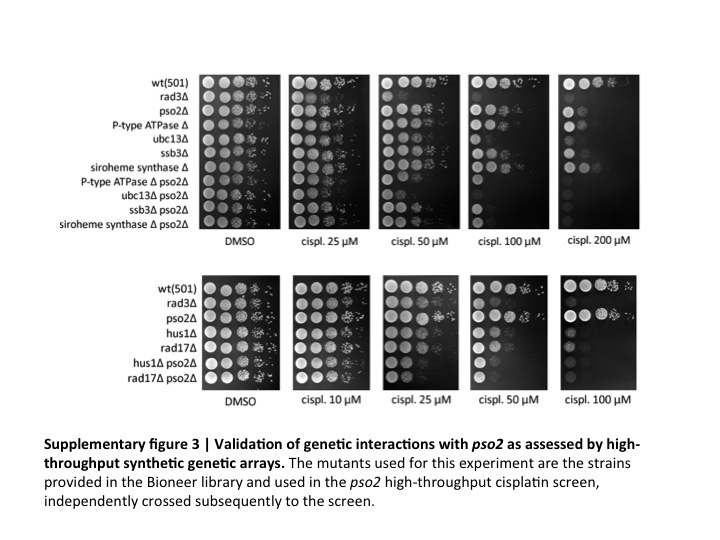


**Supplementary figure 4 | Validation of genetic interactions with *pso2* as assessed by high-throughput synthetic genetic arrays.** The mutants used for this experiment are the strains provided in the Bioneer library and used in the *pso2* high-throughput cisplatin screen, independently crossed subsequently to the screen.


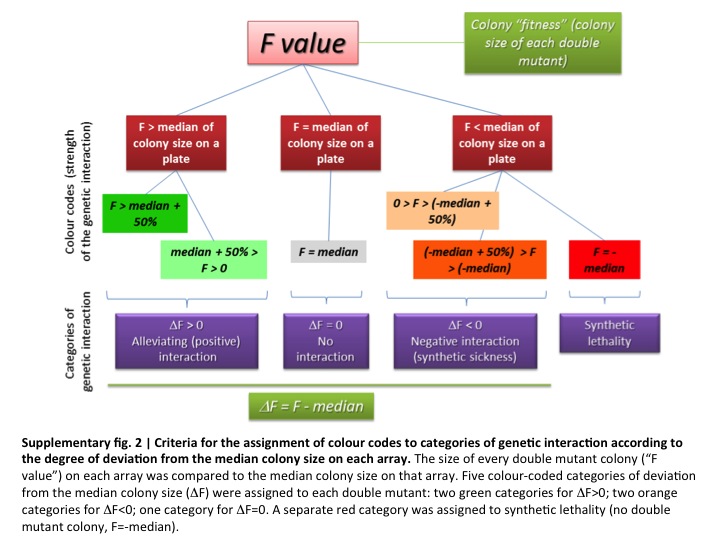


**Supplementary figure 5 | Criteria for the assignment of colour codes to categories of genetic interaction according to the degree of deviation from the median colony size on each array.** The size of every double mutant colony (“F value”) on each array was compared to the median colony size on that array. Five colour-coded categories of deviation from the median colony size (DF) were assigned to each double mutant: two green categories for DF>0; two orange categories for DF<0; one grey category for DF=0. A separate red category was assigned to synthetic lethality (no double mutant colony, F=-median).

*Intended for color reproduction on the Web only*


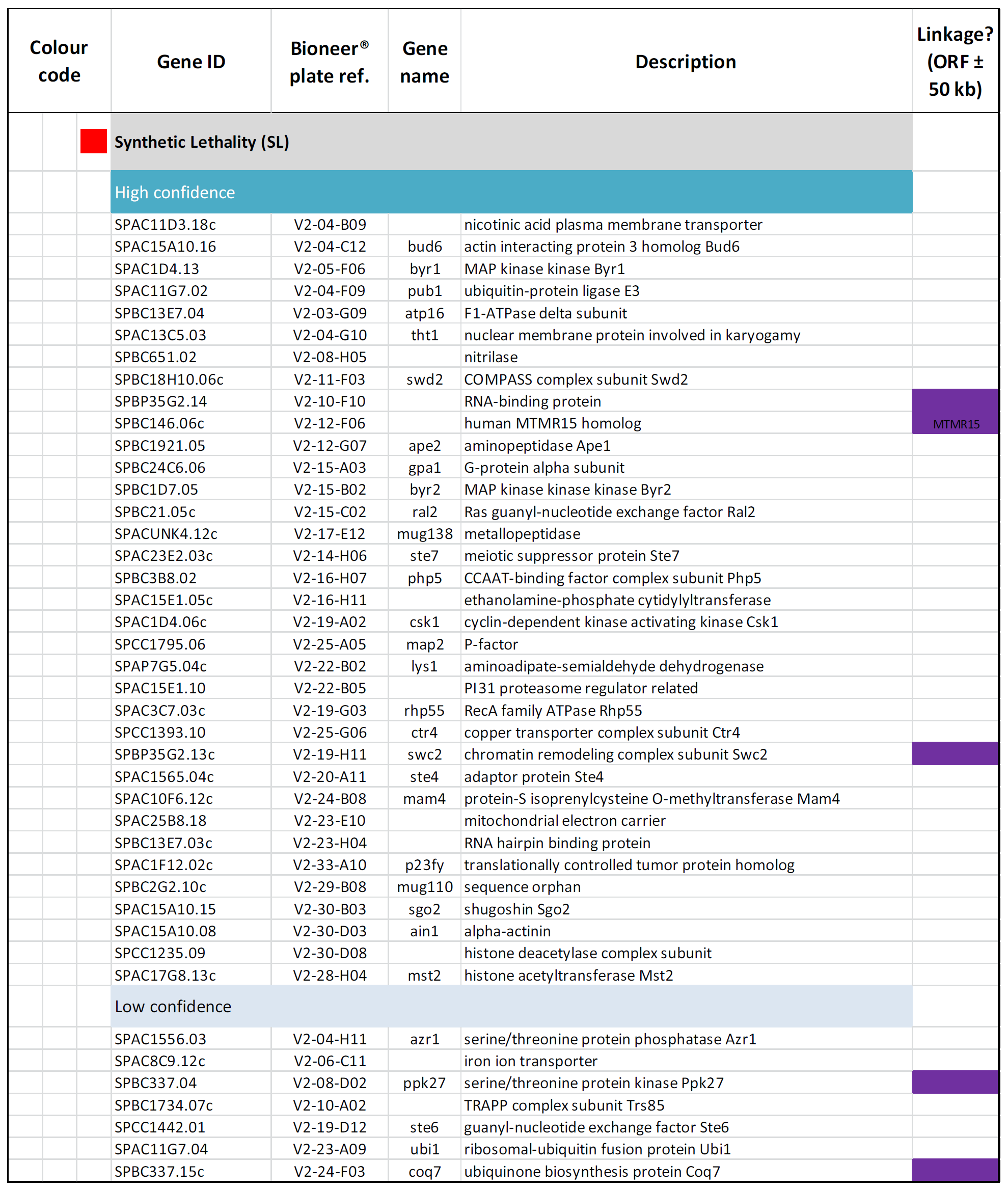


**Supplementary table 1 | Interactions between fan1 and a series of deletion mutants from the Bioneer®library showed as synthetically lethal, as determined by the computational analysis of colony size.** High confidence is defined by: 1) high consistency of results across the screens and 2) higher degree of size deviation of the double mutant from the median compared to the size deviation of the corresponding single mutant. In cases where the single mutant showed poor viability, any apparent synthetic lethality with fan1 was removed from the dataset. Gene IDs, Bioneer® plate reference, gene names and descriptions are extracted from the strain list provided with the Bioneer® deletion mutant haploid set. Linkage (ORF ± 50 kb): purple boxes indicate genes within ± 50 kb from the fan1 ORF.

*Intended for color reproduction on the Web only*


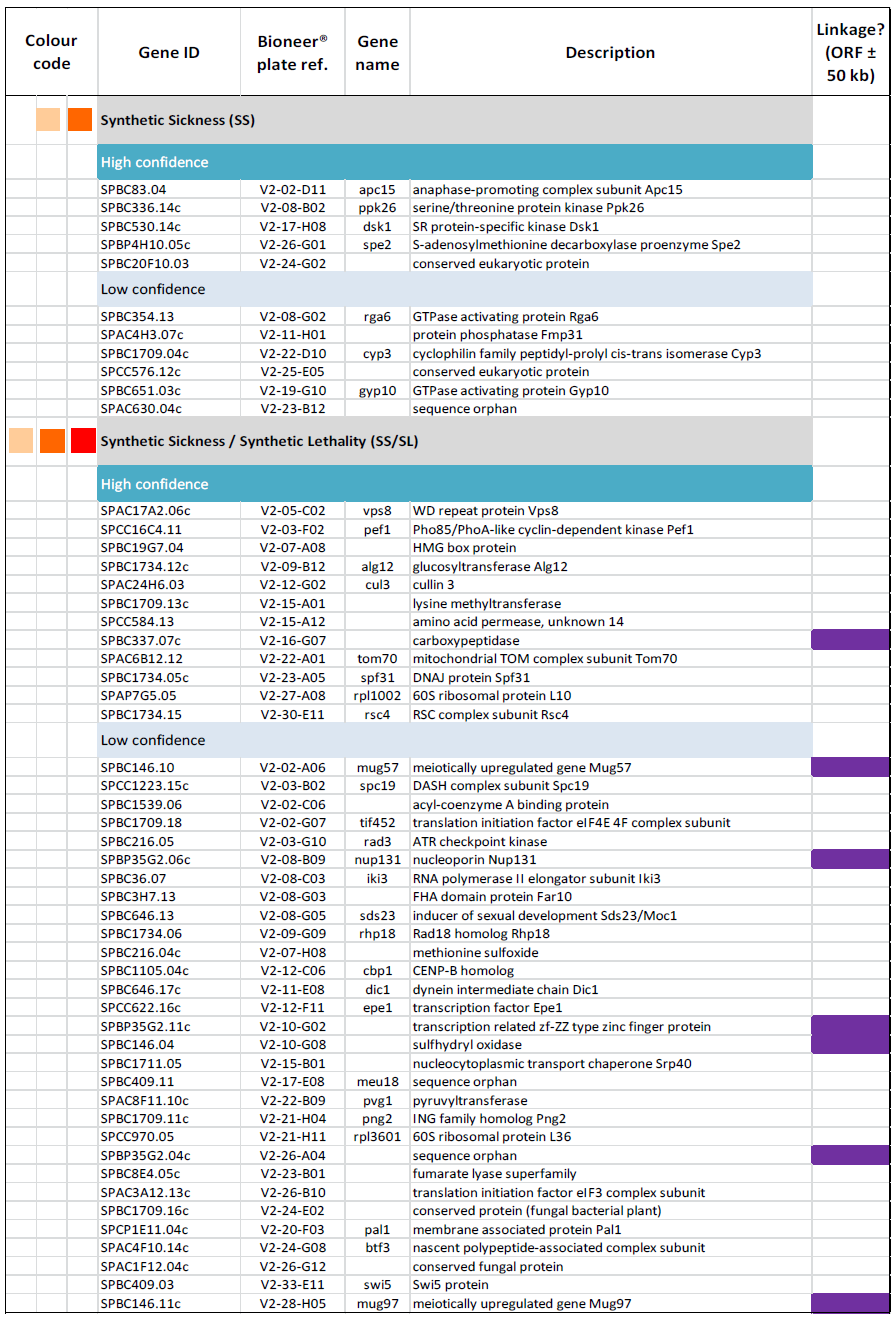


**Supplementary table 2 | Interactions between fan1 and a series of deletion mutants from the Bioneer®library showed as synthetic sick and synthetic sick / lethal (SS/SL), as determined by the computational analysis of colony size.** SS/SL represents varied but consistent negative interactions across different screens. High confidence is defined by: 1) high consistency of results across the screens and 2) higher degree of size deviation of the double mutant from the median compared to the size deviation of the corresponding single mutant. Gene IDs, Bioneer® plate reference, gene names and descriptions are extracted from the strain list provided with the Bioneer® deletion mutant haploid set. Linkage (ORF ± 50 kb): purple boxes indicate genes within ± 50 kb from the fan1 ORF.

*Intended for color reproduction on the Web only*

**
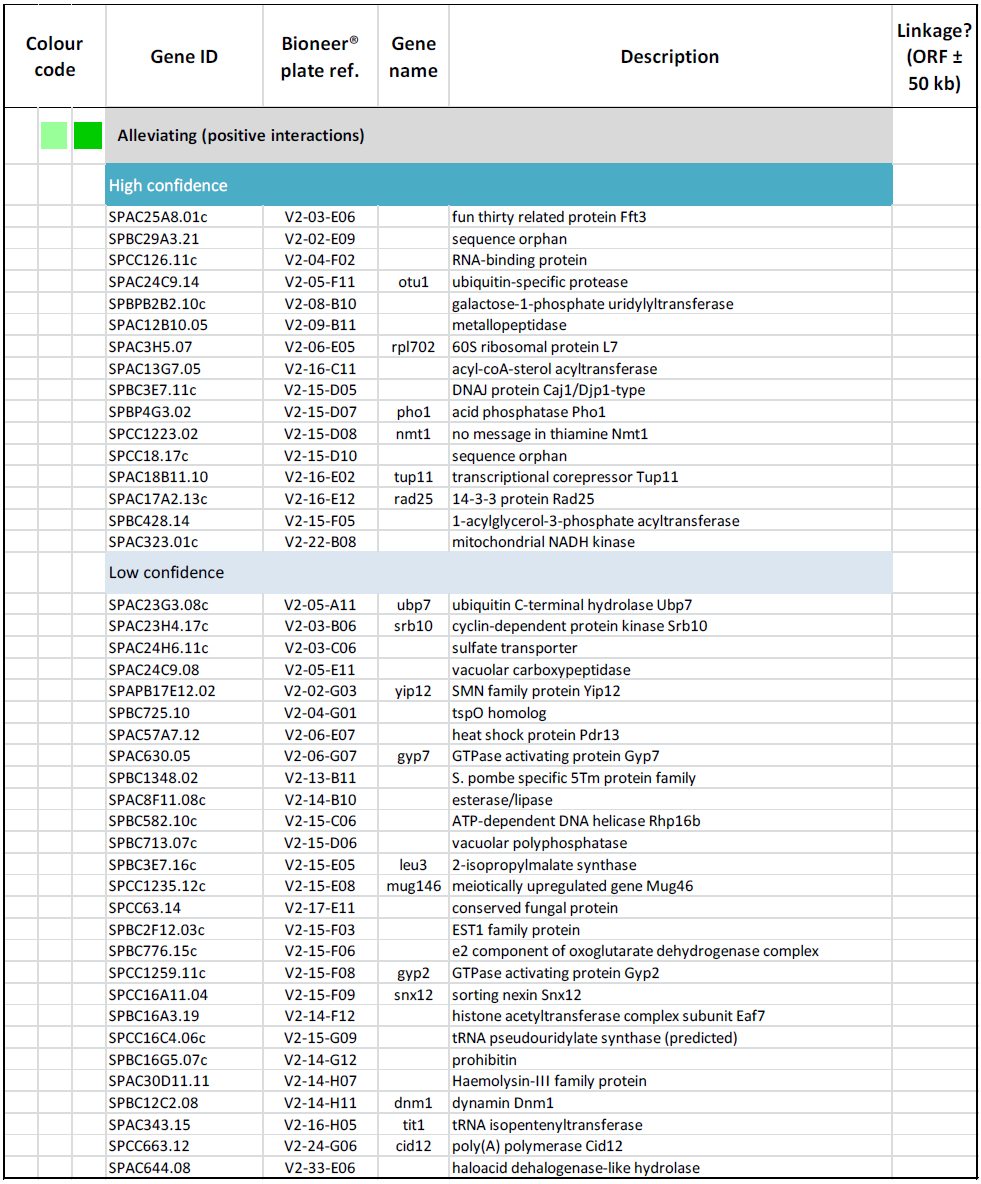
**

**Supplementary table 3 | Interactions between fan1 and a series of deletion mutants from the Bioneer®library showed as positive interactions, as determined by the computational analysis of colony size.** High confidence is defined by: 1) high consistency of results across the screens and 2) higher degree of size deviation of the double mutant from the median compared to the size deviation of the corresponding single mutant. Gene IDs, Bioneer® plate reference, gene names and descriptions are extracted from the strain list provided with the Bioneer® deletion mutant haploid set.

*Intended for color reproduction on the Web only*

*
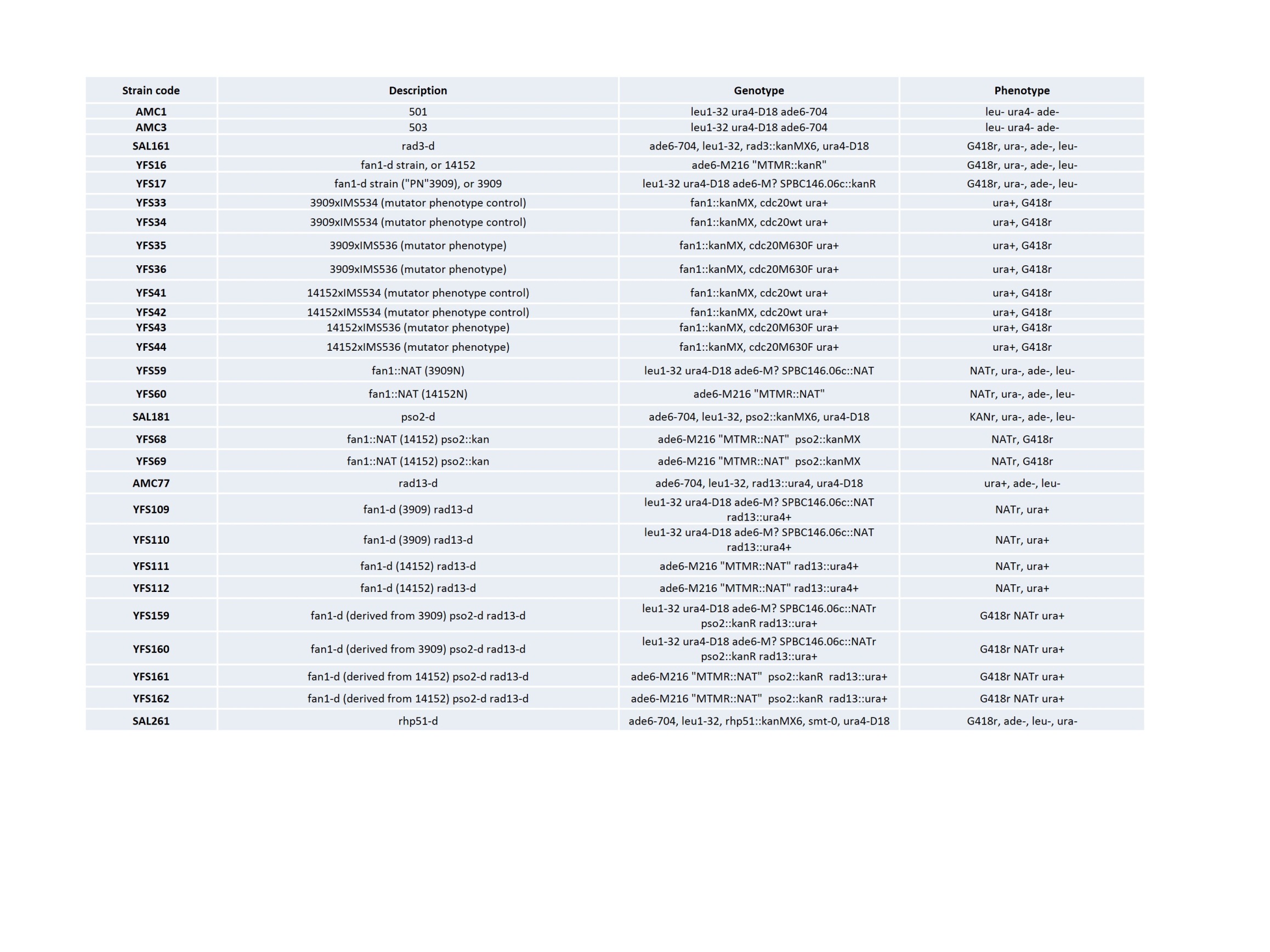
*

**Supplementary table 4 | List of strains used in this study.**


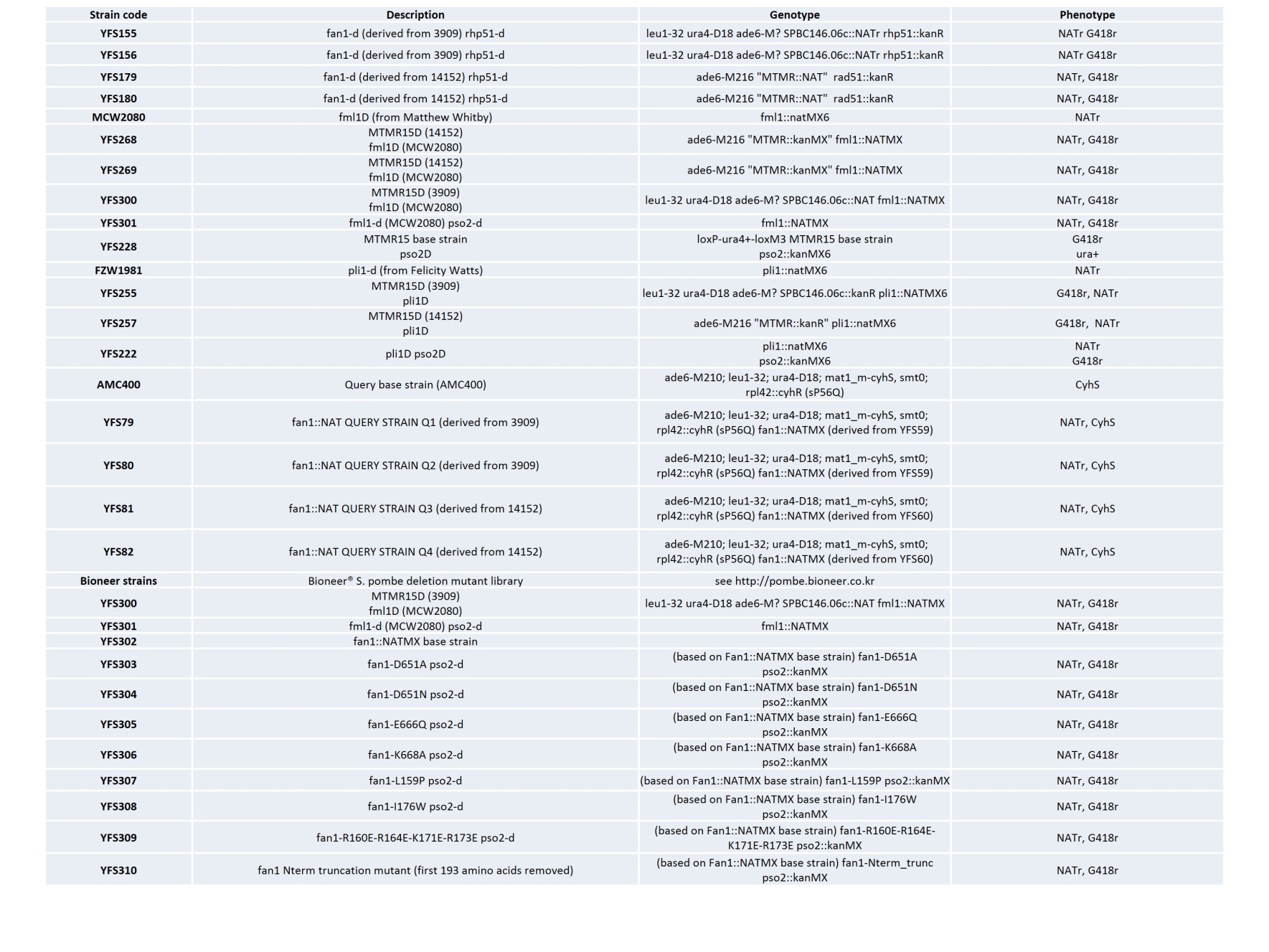


**Supplementary table 4 (continue) | List of strains used in this study.**

## Supplementary Material and Methods

**Singer® RoTor® PROGRAMS**

PROGRAM 0 (Library replicas; automatically run twice)

Use: to create replicas of the frozen gene deletion library (96 well plates)

-   WET96->WET96
-   Long pads 96
-  Program: “Library replicate” (personalised program), X# number of copies to perform
- Selected options:
-   Recycle mode: OFF
-   Revisit mode: OFF
-   Liquid mix source: ON
-   Liquid mix target: ON
- SOURCE:
- Pinning tab:
-   speed = 15 mm/sec
-   backoff = - 0.1 mm Wet mix tab:
-   diameter = 0.5 mm
-   speed = 25 mm/sec
-   cycles = 10
-   travel = 3D (0.25)
- TARGET:
- Pinning tab:
-   speed = 15 mm/sec
-   backoff = 0.3 mm Wet mix tab:
-   diameter = 1 mm
-   speed = 25 mm/se
-   cycles = 6
-   travel = 3D (3).

PROGRAM 1 (Replica WET 96 -> DRY 384)

Use: to combine deletion library 96 well plates onto agar plates (4 wet plates to 1 dry plate)

-   WET 96 -> DRY 384
-   Long pads 96
-   Program: “1 to 4 array”
- Selected options:
-   Liquid mix source: ON
-   Agar mix target: OFF
- SOURCE:
- Pinning tab:
-   speed = 15 mm/sec
-   backoff = - 0.1 mm Wet mix tab:
-   diameter = 1.3 mm
-   speed = 25 mm/sec
-   cycles = 10
-   travel = 3D (1)
- TARGET:
- Pinning tab:
-   pin pressure = 32 %
-   speed = 9mm/sec
-   overshoot = 2 mm  This program was run twice per array to ensure that enough cells are transferred.

PROGRAM 2 (BATH 96 -> DRY 384)

Use: to transfer cells from a query strain culture onto agar plates (384)

-   BATH 96 -> DRY 384
-   Long pads 96
-   Program: “1 to 4 array single source”
- Selected options:
-   Recycle mode: ON
-   Revisit mode: ON
-   Liquid mix source: ON
-   Agar mix target: OFF
- SOURCE:
- Pinning tab:
-   speed = 15 mm/sec
-   backoff = 0.4 mm Wet mix tab:
-   diameter = 1.3 mm
-   speed = 25 mm/sec
-   cycles = 5
-   travel = 3D (1)
- TARGET:
- Pinning tab:
-   pin pressure = 32 %
-   speed = 9mm/sec
-   overshoot = 2 mm  This program was run twice per array to ensure that enough cells are transferred.

PROGRAM 3 (DRY 384 -> DRY 384)

Use: to replicate colonies from agar (384) to agar plates (384), single replicas.

-   DRY 384 -> DRY 384
-   short pads 384
-   Program: “Replicate”
- Selected options:
-   Recycle mode: OFF
-   Revisit mode: OFF
-   Agar mix source: OFF (Optional: ON – see below*)
-   Agar mix target: OFF
- SOURCE:  Pinning tab:
-   pin pressure = 32 %
-   speed = 9mm/sec
-   overshoot = 2.5 mm
- *Dry mix tab:+  This mode is optional, but it might be needed to make sure to replicate also smaller, scattered colonies.
-   clearance = 2.5 mm
-   diameter = 1 mm
-   cycles = 1
- TARGET:
- Pinning tab:
-   pin pressure = 32 %
-   speed = 9mm/sec
-   overshoot = 2.5 mm

PROGRAM 4 (MATING, DRY 384 -> DRY 384)

Use: to mate colonies from agar (384) to agar plates (384).

-   DRY 384 -> DRY 384
-   short pads 384
-   Program: “mate”  Selected options:
-   No offset
-   Agar mix source: OFF (Optional: ON – see below*)
-   Agar mix target: OFF
- SOURCE:
- Pinning tab:
-   pin pressure = 32 %
-   speed = 9mm/sec
-   overshoot = 2 mm
- *Dry mix tab:+  This mode is optional, but it might be needed to make sure to replicate also smaller, scattered colonies.
-   clearance = 2.5 mm
-   diameter = 1 mm
-   cycles = 1
- TARGET:
- Pinning tab:
-   pin pressure = 32 %
-   speed = 9mm/sec
-   overshoot = 2 mm  This program was run twice.

PROGRAM 5 (Dry 384 -> Dry 384, automatically run twice)

Use: to create replicas of the 384 agar plates (intermediate steps of the screening, double replicas)

-   Dry 384 -> Dry 384
-   Short pads 384
-  Program: “Library replicate X1” (personalised program)  Selected options:
-   Recycle mode: ON
-   Revisit mode: ON (See **IMPORTANT NOTE** below)
-   Agar mix source: OFF (Optional: ON – see below)
-   Agar mix target: OFF
- SOURCE:
- Pinning tab:
-   pin pressure = 32 %
-   speed = 9mm/sec
-   overshoot = 2.5 mm
- *Dry mix tab:+  This mode is optional, but it might be needed to make sure to replicate also smaller, scattered colonies.
-   clearance = 2.5 mm
-   diameter = 1 mm
-   cycles = 1
- TARGET:
- Pinning tab:
-   pin pressure = 32 %
-   speed = 9mm/sec
-   overshoot = 2.5 mm.

**Computational analysis of colony size**

In order to detect phenotypic changes in the double mutant colonies showing a decrease (synthetic sickness) or increase (alleviating interaction) in colony size, a computational procedure has been employed which relies on the use of digital imaging analysis. Previous work has shown that colony size can be used as a phenotypic readout to assess epistatic interactions (Schuldiner et al., 2005; Schuldiner et al., 2006; Collins et al., 2006; Collins et al., 2007). The approach presented in this chapter is based on the method presented in Collins et al., 2006. Since interactions between a couple of genes in living organisms are rare (Pan et al., 2004; Tong et al., 2004; Schuldiner et al., 2005), the median colony size expected for double mutant colonies can be used as a reference to determine significant deviations that can reflect aggravating or alleviating interactions between pair or genes (Collins et al., 2006). However, due to the nature of our analysis, which is limited to a single query mutant strain, comprehensive and statistically robust approaches such as E-MAP as presented in Collins et al. (2006) would not be feasible. For this reason, the imaging software HT Colony Grid Analyser presented in Collins et al., 2006 was used to build only a semi-quantitative dataset of categories of genetic interaction between the *fan1* and *pso2* query mutants and a series of null mutant from the Bioneer® library. This procedure was applied further to the construction of SGAs (supplementary figure 3).

The first step was to extract raw colony size from digital images of single and double mutant arrays (supplementary figure 3, steps d and i respectively). For each array, the median colony size was calculated and compared to the size of every colony on the same plate. To exclude biases due to differential growth of colonies dependent on their location on the plates, separate medians were calculated for the two outermost and for the innermost rows and columns of the plate. However, when the values were compared with each other, the difference in pixels was never significantly high enough to justify a separate analysis. Thus, a single value of median colony size was used for each plate. Five colour-coded categories of deviation from the median colony size (ΔF = F value – median) were assigned according to the criteria shown in supplementary figure 5. A separate category was assigned to synthetic lethality, where F=-median.

The second step in this analysis was to collect together the data from all the independent screens on a single spreadsheet for each genetic array. The third step was to assign double mutants to categories of genetic interactions with a high or low degree of confidence, depending on criteria of consistency across different screens and healthiness of the single mutants. As our interest was to explore genetic interactions in the context of ICL repair, the computational approach presented above was applied to a screen for synthetic hypersensitivity to the DNA damaging agent cisplatin. Plates carrying double mutant strains from the automated replicas (supplementary figure 3, step i) were replicated onto plates containing increasing concentrations of cisplatin (0 micromolar, 50 micromolar, 200 micromolar, 600 micromolar). The size of the colonies arising after 24 hours and 39 hours of growth was calculated and analysed, in search for consistent and progressive reduction in colony size with increasing concentrations of cisplatin (supplementary figure 3, step l).
